# Quantification of tumour budding, lymphatic vessel density and invasion through image analysis in colorectal cancer

**DOI:** 10.1186/1479-5876-12-156

**Published:** 2014-06-01

**Authors:** Peter D Caie, Arran K Turnbull, Susan M Farrington, Anca Oniscu, David J Harrison

**Affiliations:** 1 Digital Pathology Unit, Laboratory medicine, Royal Infirmary of Edinburgh, Edinburgh EH16 4SA, UK; 2 Institute of Genetics and Molecular Medicine, University of Edinburgh, Western General Hospital, Edinburgh EH4 2XU, UK; 3 Systems Pathology, School of Medicine, University of St Andrews, St Andrews KY16 9TF, UK

**Keywords:** Image analysis, Lymphatic vessel density, Lymphatic vessel invasion, Tumour budding, Colorectal, Prognosis, Digital pathology

## Abstract

**Background:**

Tumour budding (TB), lymphatic vessel density (LVD) and lymphatic vessel invasion (LVI) have shown promise as prognostic factors in colorectal cancer (CRC) but reproducibility using conventional histopathology is challenging. We demonstrate image analysis methodology to quantify the histopathological features which could permit standardisation across institutes and aid risk stratification of Dukes B patients.

**Methods:**

Multiplexed immunofluorescence of pan-cytokeratin, D2-40 and DAPI identified epithelium, lymphatic vessels and all nuclei respectively in tissue sections from 50 patients diagnosed with Dukes A (n = 13), Dukes B (n = 29) and Dukes C (n = 8) CRC. An image analysis algorithm was developed and performed, on digitised images of the CRC tissue sections, to quantify TB, LVD, and LVI at the invasive front.

**Results:**

TB (HR =5.7; 95% CI, 2.38-13.8), LVD (HR =5.1; 95% CI, 2.04-12.99) and LVI (HR =9.9; 95% CI, 3.57-27.98) were successfully quantified through image analysis and all were shown to be significantly associated with poor survival, in univariate analyses. LVI (HR =6.08; 95% CI, 1.17-31.41) is an independent prognostic factor within the study and was correlated to both TB (Pearson r =0.71, *p* <0.0003) and LVD (Pearson r =0.69, *p* <0.0003).

**Conclusion:**

We demonstrate methodology through image analysis which can standardise the quantification of TB, LVD and LVI from a single tissue section while decreasing observer variability. We suggest this technology is capable of stratifying a high risk Dukes B CRC subpopulation and we show the three histopathological features to be of prognostic significance.

## Background

Although there exist subtypes of colorectal cancer (CRC), defined by disrupted molecular pathways, in clinical practice prognosis and recommendation for adjuvant therapy relies upon histopathological analysis of haematoxylin and eosin (H&E) stained tissue sections and the consequent TNM or Dukes staging of the tumour [1,2]. Surgical resection is undertaken as a curative procedure for Dukes A and B patients [3]. However, there is a subgroup, of 5 and 20-30% of patients respectively, who relapse and experience poor 5 year survival rates [4]. It is therefore imperative to successfully identify those patients who are at high risk of disease recurrence and who may have been under-staged.

A wealth of original studies, systematic and meta-analysis reviews have been published on the subject of lymphatic vessel invasion (LVI) [5-7], lymphatic vessel density (LVD) [8-10] and tumour budding (TB) [11-15] in CRC prognosis [16,17]. A number of these have concentrated on early stage disease and the ability to utilise histopathological features to predict lymph node metastasis or to stratify patients at high risk of disease recurrence and poor outcome [6,9,16,18-22]. Although LVI and TB have been shown to be significantly prognostic in most of these studies they are not, along with LVD, routinely reported in the clinical pathology report and are not included within the minimum core data set compiled by the Royal College of Pathologists [1], wherein only TB is listed as a non-core data item. There are a number of reasons for this; inter-observer variability [7,9,23,24], multiple quantification methodologies resulting in no agreed or standardised reporting system [12,14,15,25] and the difficulty in observing occult phenomena in routine H&E stained tissue sections [26-28]. In fact lymphatic vessels, unlike blood vessels, are especially difficult to observe in H&E stained tissue sections where they may be confused with retraction artefact. Specific stains are more frequently being employed [9,22,26,28-30] in order to increase reporting rate and decrease observer variability but there is no consensus yet on what is best. Thus the manual, semi-quantitative scoring employed in these studies is subjective, open to variability and time consuming.

The field of digital pathology is gaining momentum and beginning to be incorporated into routine clinical practice with regulatory clearance for primary diagnosis, for example, most recently approved in Canada [31]. Several studies incorporate image analysis, for example the quantification of immune infiltrate in the CRC microenvironment [32,33], and demonstrate the advantages this methodology could bring to the clinic. Automated image analysis of pathological slides, with its attributes of robust high throughput, fully quantitative and continuous data sets, however still remains largely in the realm of research.

Here we demonstrate methodology for the computer based quantification of LVI, LVD and TB at the invasive front of CRC tissue sections that could, if validated, be applied in clinical practice. The study utilises multiplexed immunofluorescence coupled with a novel image analysis algorithm to quantify the three histopathological features from a single tissue section. This approach allows implementation of standardised, high throughput quantification which could be used consistently in different institutes while minimising observer variability. We investigate if the quantification of the three histopathological features allows the stratification of Dukes B patients into high and low risk of poor outcome. Additionally we assess the potential for the methodology to further stratify patients with Dukes A to C CRC, into more precisely defined high and low risk subgroups.

## Methods

### Patients and specimens

50 patients were selected from a prospectively collected, pan-Scotland CRC cohort of patients under the age of 60 for whole slide imaging (WSI). Dukes B patients were selected on outcome (n = 29; 16 survived follow up and 13 died of CRC during follow up) in order to assess the ability of the methodology to successfully stratify the subgroup as high or low risk of disease specific survival. Dukes A (n = 13) and Dukes C (n = 8) patients were randomly selected for this study from the pan-Scotland cohort. Patients underwent surgical resection between the years of 1996 and 2003. Tumours located from the Caecum to transverse colon were amalgamated into right-sided CRC (n = 17), tumours left of the splenic flexure to the sigmoid colon were defined as left-sided CRC (n = 16) and tumours from within the recto-sigmoid and rectum were defined as rectal CRC (n = 17). A total of 40% of the patients died specifically from CRC within the patient follow up which was up to 15 years. Clinicopathological characteristics are summarised in Table 1.

**Table 1:** Clinicopathological data with univariate and multivariable analysis for disease specific survival within the CRC cohort

**Clinicopathological parameters**	**Patient number (n)**	**Univariate**				**Multivariable**			
		**HR**	**95% CI**		**P value**	**HR**	**95% CI**		**P value**
			**Lower**	**Upper**			**Lower**	**Upper**	
**Dukes**		**3.34**	**1.61**	**7.01**	**0.001**	0.33	0.08	1.36	0.12
A	13								
B	29								
C	8								
**Gender**		1.77	0.73	4.31	0.26	N/A			
M	24								
F	26								
**Age at diagnosis**		0.99	0.92	1.05	0.773	N/A			
30s	5								
40s	27								
50s	18								
**T stage**		**3.04**	**1.53**	**6.03**	**0.001**	**5.22**	**1.77**	**15.44**	**0.03**
pT1	7								
PT2	7								
pT3	27								
pT4	9								
**N Stage**		**2.62**	**1.54**	**4.46**	**<0.001**	1.62	0.69	3.81	0.27
N0	40								
N1	8								
N2	1								
N3	1								
**Differentiation**		1.14	0.37	3.54	0.83	N/A			
Well	5								
Moderate	39								
Poor	6								
**Histology**		0.49	0.11	2.11	0.34	N/A			
Standard	42								
Mucinous	8								
**Site**		0.77	0.45	1.31	0.33	N/A			
Rectal	17								
Right side	17								
Left side	16								
**Budding**		**5.76**	**2.38**	**13.8**	**0.0005**	2.56	0.9	7.27	0.08
High	13								
Medium	19								
Low	18								
**LVI**		**9.99**	**3.57**	**27.98**	**0.0001**	**6.08**	**1.17**	**31.41**	**0.03**
High	13								
Medium	19								
Low	18								
**LVD**		**5.15**	**2.04**	**12.99**	**0.00001**	1.3	0.3	5.59	0.72
High	18								
Medium	11								
Low	21								

N/A indicates that the parameter was not included for multivariable analysis due to insignificance in univariate analysis. A parameter with a hazard ratio (HR) of greater than one has an adverse effect on survival and a p value of less than 0.05 was considered significant (bold type).

The tissue used in this project was residual diagnostic material stored in the NHS Lothian diagnostic archive and provided by the NHS Lothian NRS BioResource.

The provison of this material and the subsequent research was conducted under the approval held the NHS Lothian NRS BioResource, which is a REC-approved Research Tissue Bank (REC approval ref: 13/ES/0126). This approval was granted by East of Scotland Research Ethics Service, which is part of the National Research Ethics service and NHS Health Research Authority. This provides the necessary ethical approval for the BioResource, and associated researchers to collect, store and use patient samples and associated clinical data for research. In accordance with the approval held, all samples were anonymised when released by the BioResource to the research group.

Each patient was interviewed and signed informed consent was obtained for partaking in research.

### Immunofluorescence

FFPE tissue blocks were sectioned at 4 μm, dewaxed and rehydrated in preparation for immunofluorescence. Pressure cooker heat-induced antigen retrieval was performed with Tris-EDTA, pH9 buffer. Endogenous hydrogen peroxidase was blocked with 3% hydrogen peroxide solution. Sections were incubated in DAKO serum-free block (DAKO, X0909) to reduce non-specific binding of antibodies. Next, sections were incubated for 1 hour at room temperature with primary mouse antibody against D2-40 (Dako, M3619, 1:2000) prior to an overnight 4˚C incubation of primary rabbit antibody against wide spectrum cytokeratin (Dako, Z0622, 1:150). Both antibodies were diluted in Dako antibody diluent (Dako, S0809). Dual antibody visualisation was performed by incubating slides in a secondary antibody solution of goat anti-rabbit Alexa555 conjugated antibody (Invitrogen, A21428, 1:25) and Dako Envision goat-mouse HRP antibody (Dako, K4001) for 1.5 hours in the dark at room temperature. Cy5 Tyramide (Perkin Elmer, SAT705A001EA, 1:100) was used to incubate the tissue for 10 minutes at room temperature for HRP signal amplification. Finally nuclei were visualised through slides being counterstained and mounted by adding Prolong Gold anti-fade reagent containing DAPI (Invitrogen, P36931) to a coverslip.

### Image analysis

Low resolution WSI was performed at 4x objective using an Olympus AX-51 epifluorescence microscope (Olympus, Pennsylvania, USA). The invasive front of each tissue section was visually established from the low resolution image and then captured through a series of 20x objective images. Post capture the monochromatic high definition image layers for the panCK (epithelium), D2-40 (lymphatic vessel) and DAPI (nuclei) channels were imported into Definiens image analysis software (Definiens AG, Munich) for image segmentation and classification of TB, LVI and LVD. Images were automatically segmented into the regions of interest (ROIs); Tumour, Stroma, Necrosis/Lumen and No tissue, after a previous supervised image based machine learning step utilising Definiens’ Composer Technology within Tissue Studio®. DAPI, panCK and D2-40 fluorescence were segmented utilising intensity and area thresholds and classified into Nuclei, Marker 1 and Marker 2 objects respectively. The image analysis workspace was then imported into Definiens Developer XD™ software for more sophisticated hierarchical image manipulation and object optimisation whilst negating false positive nuclei and objects. Stromal Marker 1 and Marker 2 objects and their co-localisation were then classified into tumour bud categories, lymphatic vessels and lymphatic vessel invasion, respectively, prior to their quantification. Tumour buds were defined as containing 1–5 cells only [13,34]; therefore Marker 1 objects must contain 1–5 associated nuclei to be classified as a tumour bud. The invasive front of the smallest tissue section was captured in its entirety by 15 images. Therefore only 15 images from the invasive front of subsequent tissue sections were used to quantify the histopathological features. The 15 images containing the highest number of LVI events were selected and from where LVD and TB counts were calculated.

### Data handling and statistics

Prior to statistical analysis TB and LVI objects, exported from the 15 images, were totalled while the average LVD per patient, represented as D2-40 percentage of stroma, was calculated from the 15 images. Post data handling, LVI, LVD and TB parameters were loaded into X-Tile (University of Yale) [35] software along with patient outcome information and optimal cut-offs for each parameter were calculated. Corrected *P*-values for the cut-offs were calculated using cross-validation within Monte Carlo simulations (n = 1000). Cut-offs, patient data and variables were uploaded into SPSS software for Cox-regression univariate and multivariable analysis. Pearson’s Correlation between the histopathological features was also calculated using the SPSS software and *P*-values for the correlation was adjusted by Bonferoni correction for multiple testing. TMA navigator (http://www.tmanavigator.org/) [36] was used to plot the Kaplan-Meier curves and the significance of the separation was calculated via the log-rank (Mantel-Cox) test while the *P*-values are false discovery rate (FDR) corrected using the Benjamini-Hochberg procedure to account for multiple hypothesis testing.

## Results

### Image analysis pipeline for the quantification of histopathological features (Figure 1)

**Figure 1:**
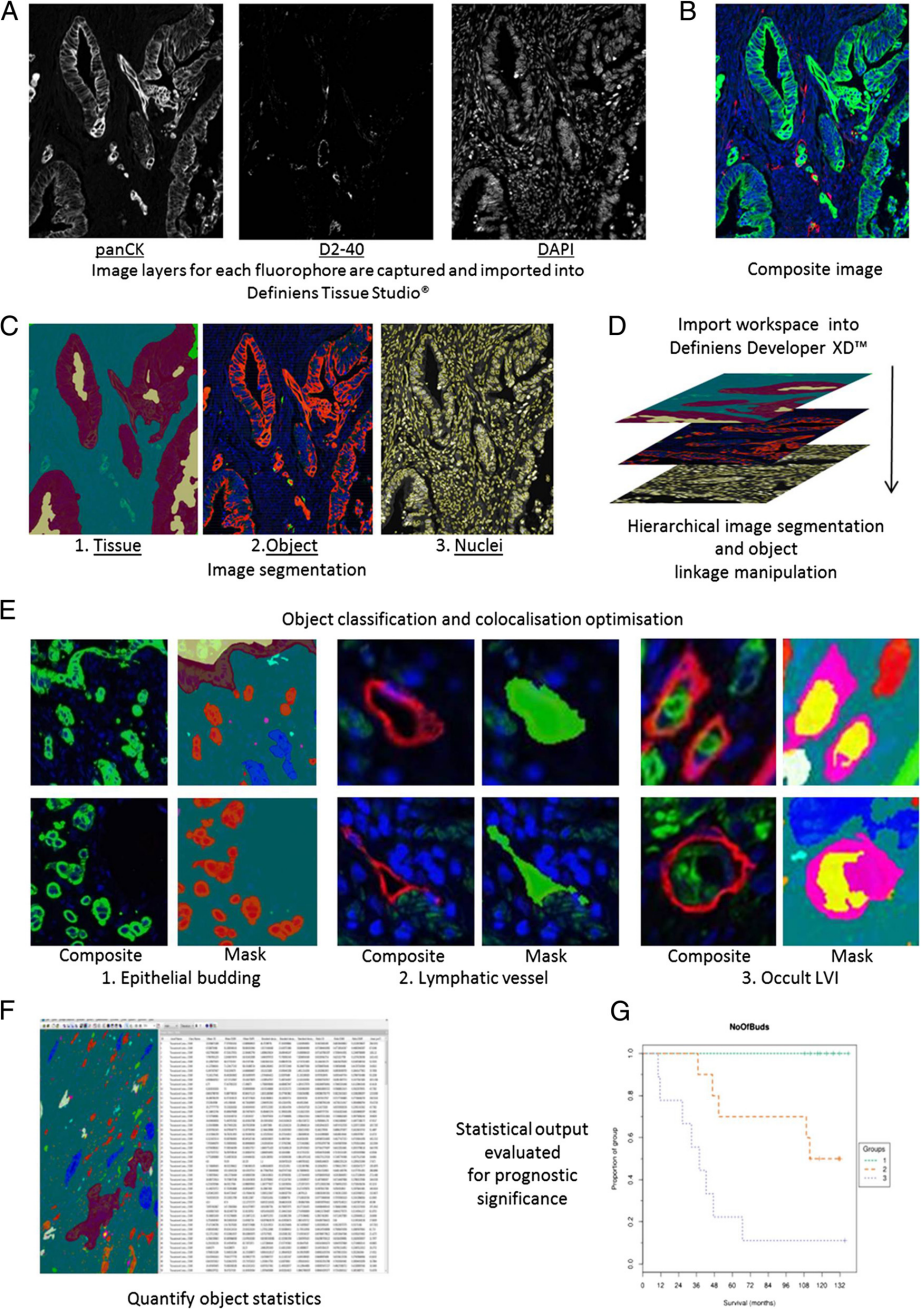
**Image analysis pipeline for histopathological feature quantification. A)** Images for each wavelength are acquired and digitised prior to being imported into Definiens Tissue Studio®. **B)** A Composite image is created within the software; green (panCK), red (D2-40) & blue (DAPI). **C)** 1. Tissue level segmentation: Tissue is segmented prior to image based machine learning through Definiens Composer technology; blue (stroma), maroon (tumour), mustard (lumen/necrosis). **C)** 2. Object level segmentation: PanCK (Marker 1: red) and D2-40 (Marker 2: green) staining above set thresholds are segmented. **C)** 3. Nucleus level segmentation: DAPI channel is used to segment nuclei (yellow). **D)** Analysis workspace is imported into Defineins Developer™ for hierarchical layer manipulation and false positive marker identification. **E)** Object classification and colocalisation optimisation. 1. Markers 1 in stroma (blue) classified as tumour bud (red; 1–5 nuclei), bud with debris nucleus (light blue; debris nuclei associated), irrelevant marker (pink; no associated nucleus) and large bud (dark blue; >5 nuclei). 2. Lymphatic vessels and lumen are segmented and classified (green). 3. Colocalisation of tumour buds and lymphatic vessels are classified (yellow; LVI), (pink; vessel border to LVI). **F**. Relevant objects are quantified and exported from the software; Figure 1F is representative of example data acquired from image object analysis. **G**. Prognostic results are calculated from the exported analysis data which was acquired from image object quantification.

#### Step 1: Image import and tissue segmentation

Images were imported into Definiens Tissue Studio® image analysis software in Tagged Image File Format (TIFF) using the software’s customized import feature which loads each of the 3 digitally captured image layers and creates a composite. The software was initially trained to segment the tissue into 4 distinct regions of interest by manually marking up areas of ‘stroma’, ‘tumour’, ‘necrosis/lumen’ and ‘no tissue’ on representative images to create a training set. The software’s Composer Technology® utilises image based machine learning, calculated from the training set, to automatically segment the ROIs on all subsequent images. The analysis rule set was programmed to only classify consequent objects within the stroma.

#### Step 2: Object segmentation

The algorithm next segmented the nuclei using DAPI intensity and morphometrics. PanCK and D2-40 fluorescence is segmented and classified as Marker 1 and Marker 2 objects respectively. Due to inter-patient marker fluorescence heterogeneity each tissue section was assessed for the intensity thresholds which would allow accurate segmentation of epithelial cells and lymphatic vessels.

#### Step 3: Object optimisation

After initial object segmentation has been carried out in Tissue Studio® the Definiens workspace is imported into the Developer XD™ software package where false nuclei and objects are negated dependent on area, intensity and texture. Nuclear objects which are under 16 μm^2^ are re-classified as debris nuclei. Due to the phenomenon of non-specific staining at the edge of tissue through immunohistochemistry both positive Marker 1 and 2 objects within 50 μm of ‘no tissue’ are classified as edge effect (Additional file 1: Figure S1). Remaining neighbouring Markers 1 (panCK), in the stroma ROI alone, are merged and optimised for accurate epithelial segmentation and are hierarchically classified as ‘irrelevant marker’ (no associated nuclei), ‘bud with debris nucleus’ (only debris nuclei associated with marker), ‘tumour bud’ (1–5 associated nuclei) and ‘large bud’ (over 5 nuclei associated). Neighbouring Markers 2 (D2-40), in the stroma, are also merged, while the vessel lumen is combined with the vessel wall, resulting in objects classified as vessels. Finally the co-localisation of tumour buds and vessels are classified as LVI. The number of all objects, their colocalisation and the vessel percentage of stroma (LVD) are quantified and exported to assess their prognostic relevance in stratifying CRC patients into high and low risk subpopulations.

#### Clinicopathological and Cox-regression analysis

**Figure 2:**
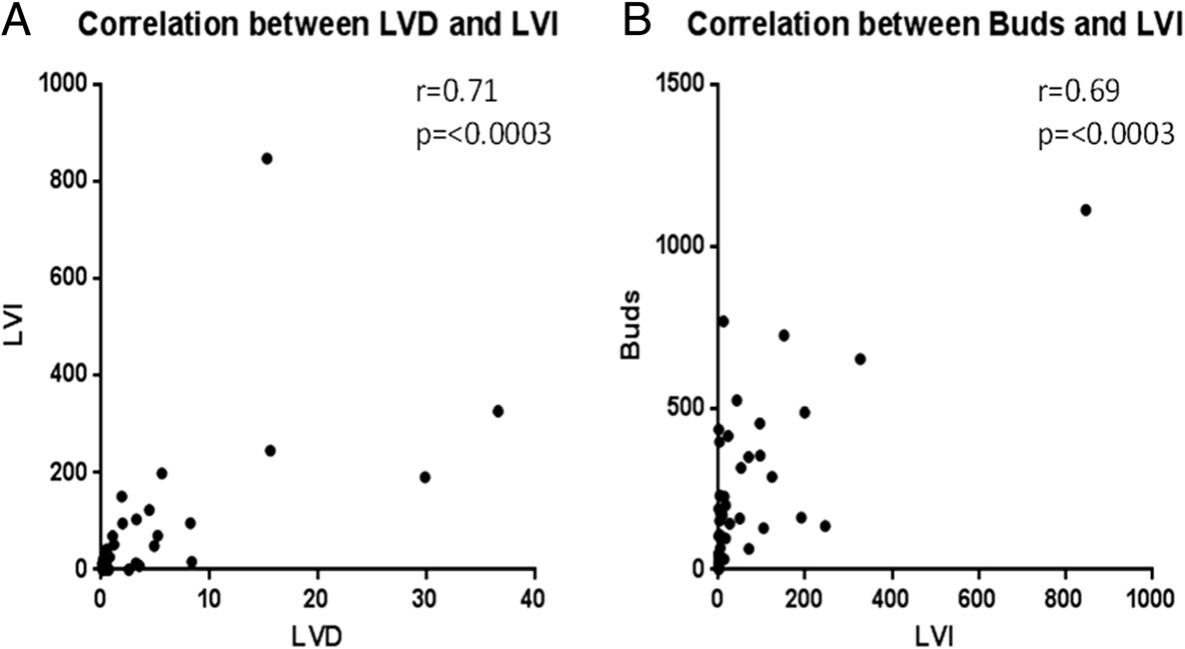
**Correlation of histopathological features.** Continuous data plotted through Pearson’s Correlation (r value) after Bonferoni correction to assess correlation between **A)** LVI and LVD and **B)** LVI and TB. Significance shown by P value.

Cut-offs were calculated from the Dukes A-C cohort for high and low sub-groups and their significance established by Monte Carlo simulations for LVI (cut-off = 16 LVI events across 15 images, *p* <0.0001), LVD (cut-off = 0.7 vessel percentage of stroma averaged across 15 images, *p* = 0.002) and TB (cut-off = 287 tumour buds across 15 images, *p* = 0.0001). The cut-offs established in this manner were then applied to stratify the Dukes B subpopulation. Clinicopathological data and regression analysis is summarised in Table 1. Univariate analysis showed TB (HR =5.7; 95% CI, 2.38-13.8), LVI (HR =9.9; 95% CI, 3.57-27.98) and LVD (HR =5.1; 95% CI, 2.04-12.99) to be significant predictors of survival within the cohort. In multivariable cox-regression analysis the predictive model was adjusted for T stage, N stage, Dukes stage, TB, LVI and LVD and showed that only depth of local invasion (T stage, HR = 5.22; 95% CI,1.77-15.44) and LVI (HR =6.08; 95% CI, 1.17-31.41) were independent predictors of survival. To assess if LVD and LVI were associated we performed Pearson’s correlation and found them to be significantly correlated (r = 0.71, *p* <0.0003). Similarly LVI and TB were also found to be significantly correlated (r = 0.69, *p* <0.0003) (Figure 2).

#### Kaplan-Meier survival curves for tumour budding

**Figure 3:**
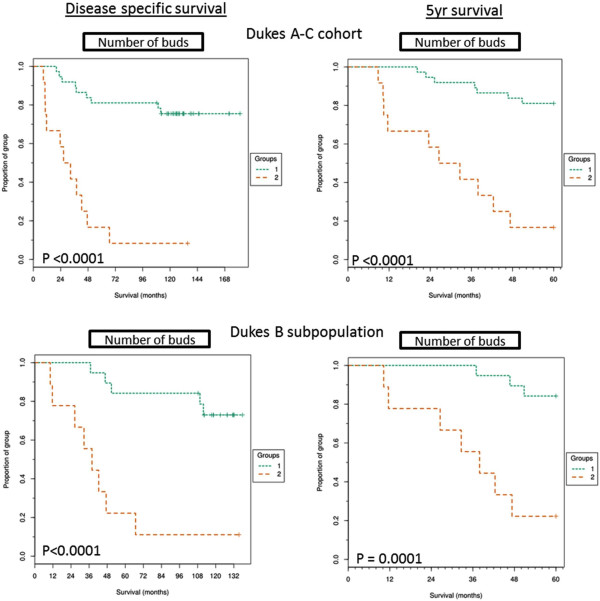
**Kaplan-Meier curves for tumour budding.** Kaplan-Meier curves showing full follow up disease specific and 5 year disease specific survival of above cut-off TB group (>287 buds, group 2) and below cut-off TB group (<287 buds, group 1) within the full Dukes A-C cohort and the Dukes B subpopulation and across disease specific survival or 5 year survival. Significance shown by P value calculated from mantel-cox analysis and FDR corrected.

KM curves were plotted using TMA Navigator to assess the prognostic relevance of tumour budding in the Dukes A to C population as well as across the Dukes B subpopulation alone (Figure 3). Tumour budding was significantly prognostic for poor outcome and shorter disease specific survival times in both the full Dukes A-C cohort (*p* <0.0001) and within the Dukes B (*p* = 0.0005) subpopulation. The percentage of patients still alive at the end of the study and in the above cut-off subgroup (>than 287 buds) was 7.7% compared to 76% in the low budding subgroup for the Dukes A-C cohort and 10% compared to 73% in the Dukes B subpopulation. Tumour budding was also found to be significant when stratifying high and low risk patients in 5 year survival rates for the full Dukes A-C cohort (*p* < 0.0001 ) and the Dukes B subpopulation (*p* = 0.0001 ). Automated analysis allows the quick comparison of the size of the tumour bud to the significance of prognosis (Additional file 2: Figure S2). The study showed there was no difference on the proportion of patients alive after full follow up in above cut-off groups when quantifying TB with 1–2, 1–5 or >5 associated nuclei or when summing tumour buds (1–5 nuclei) and large buds (>5 associated nuclei) within the Dukes B subpopulation. The quantification of different size categories of tumour buds within the full cohort also showed low percentages of patients in all categories (Table 2).

**Table 2:** Number of patients alive after full follow up in high cut-off groups within tumour bud size categories

**Patient cohort**		**Patients alive post follow up**		
	**Tumour buds (1–5 nuc)**	**Small Buds (1–2 nuc)**	**Large buds (>5nuc)**	**Total tumour budding**
Dukes A-C cohort	n = 1 (7%)	n =1 (7%)	n = 4 (23%)	n =2 (14%)
Dukes B subpopulation	n = 1 (10%)	n = 1 (10%)	n = 1 (10%)	n = 1 (10%)

The proportion of patients, after the study was complete, who were alive within the high-cut off groups of differing sizes of tumour bud categories. Total tumour budding indicates the sum total of the objects within the tumour buds and large buds categories.

### Kaplan-Meier survival curves for lymphatic vessel density

**Figure 4:**
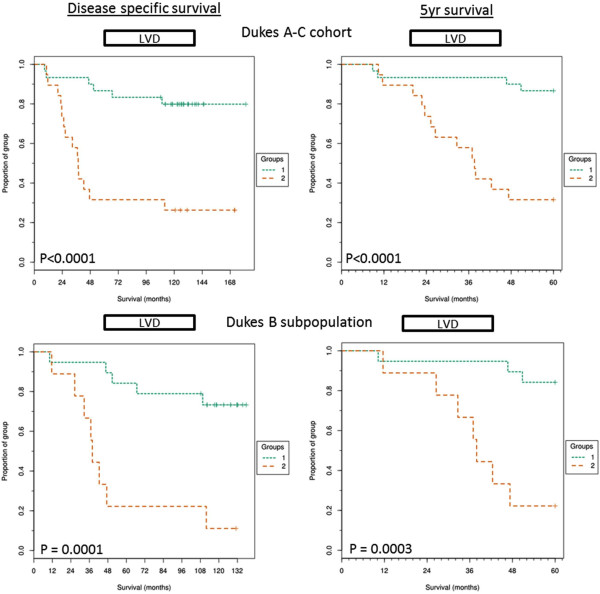
**Kaplan-Meier curves for Lymphatic vessel density.** Kaplan-Meier curves showing full follow up disease specific and 5 year disease specific survival of above cut-off LVD group (>0.7% vessels of total stroma area, group 2) and below cut-off LVD group (<0.7 vessel% of stroma area, group 1) within the full Dukes A-C cohort and the Dukes B subpopulation and across disease specific survival or 5 year survival. Significance shown by P value calculated from mantel-cox analysis and FDR corrected.

The LVD was calculated for each image, meaned across 15 images captured per patient and KM curves were plotted from the results (Figure 4). LVD was significantly associated with poor outcome and shorter disease specific survival in both Dukes A-C (*p* = 0.0001) cohort and the Dukes B subpopulation (*p* = 0.0001). Only 26% of patients within the Dukes A-C cohort and 11% within the Dukes B subpopulation, who were within the above-cut-off LVD (>0.7% vessel density) group, survived full follow up. All 8 Dukes C patients were within the high LVD subgroup. Higher LVD was also significantly associated with poor disease specific 5 year survival times for both the full cohort (*p* < 0.0001) and the Dukes B subgroup (*p* = 0.0003).

### Kaplan-Meier survival curves for lymphatic vessel invasion

**Figure 5:**
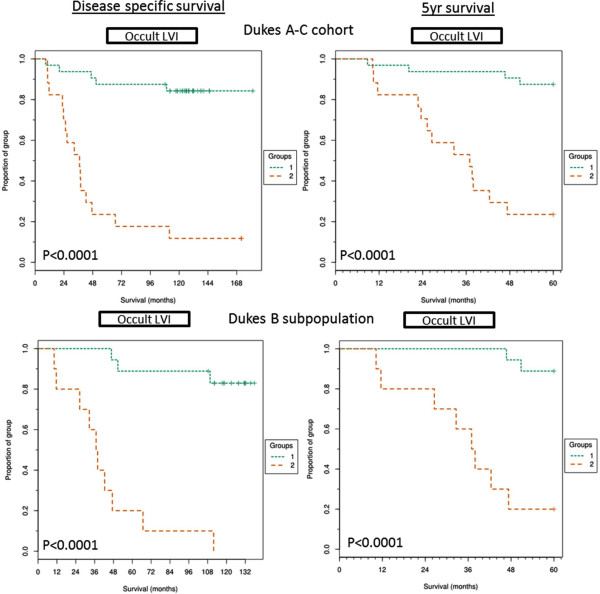
**Kaplan-Meier curves for lymphatic vessel invasion.** Kaplan-Meier curves showing full follow up disease specific and 5 year disease specific survival of above cut-off LVI group (>16 LVI events, group 2) and below cut-off LVD group (<16 LVI events, group 1) within the full Dukes A-C cohort or the Dukes B subpopulation and across disease specific survival or 5 year survival. Significance shown by P value calculated from mantel-cox analysis and FDR corrected.

Co-localisation of tumour buds and lymphatic vessels was categorised as LVI. KM analysis was performed and LVI was shown to be the most significantly prognostic parameter associated with shorter survival times in both the Dukes A-C cohort (p <0.0001) and the Dukes B subpopulation (*p* <0.0001) (Figure 5). In fact, no patients survived full follow up within the above cut-off LVI group (>16 LVI events) in the Dukes B patient subpopulation and only 11% survived follow up within the full cohort. Similarly to the other histopathological features quantified, LVI was associated with poor 5 year survival times for the full cohort (*p* < 0.0001) and the Dukes B subpopulation (*p* < 0.0001).

## Discussion

We report a novel semi-automated methodology to reliably identify and quantify three prognostic histopathological features; LVI, LVD and TB, across the invasive front of colorectal carcinoma. The features are captured and exported from a single tissue section using the one continuous image analysis algorithm. This saves considerable resource, compared to serial sectioning and staining prior to manual semi-quantification of each histopathological feature, making the approach amenable to a time-dependent clinical setting. All three histopathological features were found to be significant in predicting poor outcome and were associated with shorter survival while LVI was found to be independently prognostic. This may allow further stratification of a subgroup of Dukes B patients into low and high risk of poor outcome.

Ueno and Hase et al. [13,34] proposed the definition of a tumour bud as 1–5 undifferentiated cancer cells disseminated from the invasive edge. The majority of researchers in the field have adopted this cut-off for TB size, and have shown TB to be prognostically significant. However there is no formally agreed quantification methodology [12,15] resulting in variability in reporting [23]. As a result TB scoring has not been incorporated into the core minimal dataset and is not routinely reported in the NHS clinic [1]. TB quantification methodology with higher inter-observer concordance has been proposed by Horcic et al. [24] where TB is manually counted within 10 fields at x40 objective at the invasive front. Our automated methodology allows the quantification of TB at x20 objective across 15 fields captured at the invasive front. The 15 images used for automated TB quantification results in a larger sampling area than other studies have so far utilised [15]. Although sampling methodology for the quantification of TB differs, the definition of a tumour bud comprising only 1–5 cells remains a constant in the literature [15]. The image analysis algorithm developed for this study exports the number of nuclei associated with each tumour bud. This allows the researcher to quickly assess the impact which changing the TB size criterion has on prognosis. We show that quantifying tumour buds comprised of only 1–2 cells, tumour buds comprised of 1–5 cells and tumour buds larger than 5 cells are all individually associated with poor outcome and that the classical definition of a tumour bud is therefore a sound one.

Both blood and lymphatic vessel invasion are associated with nodal metastasis and poor prognosis [7]. Blood and lymphatic vascular invasion are, however, under-recognised in H&E staining alone [26,27]. Therefore the use of specific histochemical markers, such as Elastica staining have been employed to highlight blood vessels and increase reporting rates of invasion events [26]. LVI is difficult to confidently recognise in H&E stained CRC tissue sections. This is due to the lack of a surrounding rim of muscle, such as is found with blood vessels, and confusion of lymphatic vessels with retraction artefact. This, alongside no standardised reporting methodology, is another contributing factor for the absence of lymphatic vascular based prognostic features from the minimal core data set and so standard practice is to not report LVI or LVD in the clinic. To overcome the problem of identifying LVI, studies have employed immunohistochemical staining with a D2-40 antibody which specifically binds to lymphatic vessel endothelial cells [22,28]. Dual staining of epithelium and vessels allows easier recognition and reporting of LVI events [28] within the complex tumour microenvironment while automated quantification adds further robustness to the data. LVI alone has been associated with lymph node metastasis (LNM) and poor outcome [22,29]. The under-recognition of LVI may be a contributing factor for the under-staging of CRC patients [22,27] and disease recurrence in ~30% of the Dukes B population [4,37]. Our methodology quantifies tumour buds invading small lymphatic vessels which we term as LVI. We observe that out of all the histopathological features we measured, LVI is the most significantly prognostic. KM analysis shows that no Dukes B patients with above cut-off LVI survived follow up. LVI was also the only parameter to independently be associated with an adverse effect on disease specific survival. Some studies have shown LVD to be prognostic and associated to poor outcome or LNM [8,38] however others show that no correlation exists [9,39]. LVD assessment is, however, not standardised and most researchers employ various magnifications and numbers of LVD “hot-spots” utilised to create a mean LVD while observer variability has been shown to be strong [9]. Bias is therefore introduced to these studies which can be negated by WSI and automated image analysis of the invasive front or entire tissue section. In the methodology, which we demonstrate here, we automatically segment the stroma from the tumour and by doing so we are able to calculate the LVD only within the stromal compartment. The methodology does not rely on the manual locating of LVD hot-spots; rather the quantification of LVD across 15 images taken from the invasive front which contained the highest LVI events is used. By adopting this LVD calculation and minimising sampling bias we have shown LVD to be significantly associated to poor outcome (p = 0.0001). All Dukes C patients had a high category of LVD which suggests that lymphangiogenesis may occur as the disease progresses.

It is unknown whether lymphangiogenesis occurs due to a host reaction attacking the tumour or by tumour cell signalling, however we show that LVI is correlated to LVD (r = 0.71, p < 0.0003) which is in accord with separate studies [40,41]. We also observed that LVI is associated with TB (<0.0003), as did Ohtsuki et al. [28]. TBs could be suggested to be a more invasive subpopulation of cells disseminated from the tumour mass and if so may have acquired the ability to invade the lymphatic system and metastasise to distant nodes.

The automatic quantification of prognostic histopathological features lends further proof to the value of reporting TB, LVI and LVD to stratify high risk CRC patients. The methodology is amenable to standardisation between institutions allowing consistent reporting of CRC. TB, LVI and LVD were all more significantly associated with poor outcome than Dukes staging, when performing univariate regression analysis, within this proof of methodology study. The image analysis quantification methodology of these three histopathological features, upon further validation in large and disparate cohorts, may become more widely accepted as standardised prognostic factors amenable to being incorporated into the minimal core data set and TNM staging.

The ability to quantify prognostically relevant histopathological features, in a robust and routine manner through automated image analysis, will not only standardise the practice and negate observer variability but will free up a pathologist’s valuable time. We believe that as digital pathology becomes more common place within the clinic, automated quantification of histopathological features, as demonstrated here, will become an invaluable tool in the pathologist’s repertoire to stratify high risk cancer patients.

## Conclusion

In conclusion, we demonstrate a computer based image analysis methodology to quantify tumour buds, lymphatic vessel density and lymphatic vessel invasion in immunofluorescently labelled colorectal cancer tissue sections. This methodology has the means to standardise the quantification of the three histopathological features in a robust fashion. We applied the methodology to a colorectal cancer cohort consisting of patients spanning Dukes A –C diagnoses and found all the histopathological features to be significantly relevant to prognosis. Lymphatic vessel invasion, in our study, was shown to be an independent predictor of survival.

## Abbreviations

CRC: Colorectal cancer; LVI: Lymphatic vessel invasion; LVD: Lymphatic vessel density; TB: Tumour budding; panCK: Pan cytokeratin.

## Competing interest

The authors declare that they have no competing interests

## Authors’ contributions

PDC performed all lab work, image analysis, data manipulation and wrote the manuscript. AKT performed the statistical analysis. SMF provided patient clinicopathology and follow up data. AO and DJH provided guidance, pathology support and participated in the drafting of the manuscript. All authors read and approved the final manuscript.

## Supplementary Material

Additional file 1: Figure S1 Classification of false nuclei and objects. Original composite images are pseudocoloured blue (DAPI), green (panCK) and red (D2-40). Images for the DAPI channel alone are greyscale. A)1. False nuclei are classified and negated as are nuclear debris. To negate a false nuclei count, post-segmentation, in the stroma or within tumour buds, nuclei under 16 μm^2^ are classified as debris nuclei. A white arrow shows a small section from a stromal nucleus being segmented by the tumour bud and classified as debris nucleus. A high background intensity of DAPI, increased by a segmented section of a stromal nucleus within the tumour bud, has created a false nucleus (pink). This is classified as such and negated. A)2. The tissue section in the example has high auto-fluorescence within the DAPI channel, the false nuclei segmented as a result of this are classified as such (pink). A)3. High DAPI channel auto-fluorescence of goblet cells within colonic crypts result in false nuclei segmentation, these are too classified as such (pink). B) Auto-fluorescence of muscle cells, within the Cy3 channel, may lead to their segmentation as panCk positive epithelial cells. Intensity and texture parameters are utilised to classify the falsely segmented objects as ‘non-specific CK’. C) Non-specific staining of both panCk and D2-40 antibodies occurs close to the edge of tissue. All positively segmented objects which are 50 μm from ‘no tissue’ (green) are classified as ‘edge effect’ (blue). Click here for file

Additional file 2: Figure S2 Kaplan-Meier plots comparing prognostic significance of quantifying differing sizes of tumour bud. A) Kaplan-Meier curve showing disease specific survival times for below cut-off (group 1) and above cut-off (group 2) in tumour buds with 1–5 nuclei associated. B) Kaplan-Meier curve showing disease specific survival times for below cut-off (group 1) and above cut-off (group 2)in tumour buds with 1–2 nuclei associated. C) Kaplan-Meier curve showing disease specific survival times for below cut-off (group 1) and above cut-off (group 2) in tumour buds with greater than 5 nuclei associated. D) Kaplan-Meier curve showing disease specific survival times for below cut-off (group 1) and above cut-off (group 2) upon the summing of tumour buds with 1–5 nuclei and tumour buds with greater than 5 nuclei. Click here for file

## References

[B1] Standards and Datasets for Reporting Cancers Dataset for colorectal cancer 2007 2 [http://www.rcpath.org/Resources/RCPath/Migrated%20Resources/Documents/G/G049-ColorectalDataset-Sep07.pdf]

[B2] Lan YT Yang SH Chang SC Liang WY Li AF Wang HS Jiang JK Chen WS Lin TC Lin JK Analysis of the seventh edition of American Joint Committee on colon cancer staging Int J Colorectal Dis 2012 27 657 663 22146786 10.1007/s00384-011-1366-6

[B3] Poston GJ Tait D O'Connell S Bennett A Berendse S Diagnosis and management of colorectal cancer: summary of NICE guidance BMJ 2011 343 d6751 22074710 10.1136/bmj.d6751

[B4] O'Connell JB Maggard MA Ko CY Colon cancer survival rates with the new American Joint Committee on Cancer sixth edition staging J Natl Cancer Inst 2004 96 1420 1425 15467030 10.1093/jnci/djh275

[B5] Huh JW Lee JH Kim HR Kim YJ Prognostic significance of lymphovascular or perineural invasion in patients with locally advanced colorectal cancer Am J Surg 2013 206 758 763 23835209 10.1016/j.amjsurg.2013.02.010

[B6] Chang S-C Lin C-C Wang H-S Yang S-H Jiang J-K Lan Y-T Lin T-C Li AF-Y Chen W-S Lin J-K Lymphovascular invasion determines the outcome of stage I colorectal cancer patients Formosan J Surgery 2012 45 141 145

[B7] Betge J Pollheimer MJ Lindtner RA Kornprat P Schlemmer A Rehak P Vieth M Hoefler G Langner C Intramural and extramural vascular invasion in colorectal cancer: prognostic significance and quality of pathology reporting Cancer 2012 118 628 638 21751188 10.1002/cncr.26310

[B8] Barresi V Reggiani-Bonetti L Di Gregorio C De Leon MP Barresi G Lymphatic vessel density and its prognostic value in stage I colorectal carcinoma J Clin Pathol 2011 64 6 12 20947870 10.1136/jcp.2010.083550

[B9] Cacchi C Arnholdt HM Jahnig H Anthuber M Probst A Oruzio DV Markl B Clinical significance of lymph vessel density in T3 colorectal carcinoma Int J Colorectal Dis 2012 27 721 726 22228115 10.1007/s00384-011-1373-7

[B10] Royston D Jackson DG Mechanisms of lymphatic metastasis in human colorectal adenocarcinoma J Pathol 2009 217 608 619 19253334 10.1002/path.2517

[B11] Betge J Kornprat P Pollheimer MJ Lindtner RA Schlemmer A Rehak P Vieth M Langner C Tumour budding is an independent predictor of outcome in AJCC/UICC stage II colorectal cancer Ann Surg Oncol 2012 19 3706 3712 22669453 10.1245/s10434-012-2426-z

[B12] Karamitopoulou E Zlobec I Kolzer V Kondi-Pafiti A Patsouris ES Gennatas K Lugli A Proposal for a 10-high-power-fields scoring method for the assessment of tumour budding in colorectal cancer Mod Pathol 2013 26 295 301 23018875 10.1038/modpathol.2012.155

[B13] Ueno H Murphy J Jass JR Mochizuki H Talbot IC Tumour 'budding' as an index to estimate the potential of aggressiveness in rectal cancer Histopathology 2002 40 127 132 11952856 10.1046/j.1365-2559.2002.01324.x

[B14] Mitrovic B Schaeffer DF Riddell RH Kirsch R Tumour budding in colorectal carcinoma: time to take notice Mod Pathol 2012 25 1315 1325 22790014 10.1038/modpathol.2012.94

[B15] Lugli A Karamitopoulou E Zlobec I Tumour budding: a promising parameter in colorectal cancer Br J Cancer 2012 106 1713 1717 22531633 10.1038/bjc.2012.127 PMC3364122

[B16] Mou S Soetikno R Shimoda T Rouse R Kaltenbach T Pathologic predictive factors for lymph node metastasis in submucosal invasive (T1) colorectal cancer: a systematic review and meta-analysis Surg Endosc 2013 27 2692 2703 23392988 10.1007/s00464-013-2835-5

[B17] Glasgow SC Bleier JI Burgart LJ Finne CO Lowry AC Meta-analysis of histopathological features of primary colorectal cancers that predict lymph node metastases J Gastrointest Surg 2012 16 1019 1028 22258880 10.1007/s11605-012-1827-4

[B18] Uribarrena AR Ortego J Fuentes J Raventos N Parra P Uribarrena ER Prognostic value of microvascular density in dukes a and B (t1-t4, n0, m0) colorectal carcinomas Gastroenterol Res Pract 2009 2009 679830 19902004 10.1155/2009/679830 PMC2774471

[B19] Suzuki A Togashi K Nokubi M Koinuma K Miyakura Y Horie H Lefor AT Yasuda Y Evaluation of venous invasion by Elastica van Gieson stain and tumour budding predicts local and distant metastases in patients with T1 stage colorectal cancer Am J Surg Pathol 2009 33 1601 1607 19574884 10.1097/PAS.0b013e3181ae29d6

[B20] Doyle B Hagan S Al-Mulla F Scott L Harden S Paul J Mulcahy H Murray GI Sheahan K O'Sullivan J Kolch W Raf kinase inhibitor protein expression combined with peritoneal involvement and lymphovascular invasion predicts prognosis in Dukes' B colorectal cancer patients Histopathology 2013 62 505 510 23460950 10.1111/his.12014

[B21] Betge J Kornprat P Pollheimer MJ Lindtner RA Schlemmer A Rehak P Vieth M Langner C Tumour Budding is an Independent Predictor of Outcome in AJCC/UICC Stage II Colorectal Cancer Ann Surg Oncol 2012 19 3706 3712 22669453 10.1245/s10434-012-2426-z

[B22] Barresi V Reggiani Bonetti L Vitarelli E Di Gregorio C Ponz De Leon M Barresi G Immunohistochemical assessment of lymphovascular invasion in stage I colorectal carcinoma: prognostic relevance and correlation with nodal micrometastases Am J Surg Pathol 2012 36 66 72 21989343 10.1097/PAS.0b013e31822d3008

[B23] Puppa G Senore C Sheahan K Vieth M Lugli A Zlobec I Pecori S Wang LM Langner C Mitomi H Nakamura T Watanabe M Ueno H Chasle J Conley SA Herlin P Lauwers GY Risio M Diagnostic reproducibility of tumour budding in colorectal cancer: a multicentre, multinational study using virtual microscopy Histopathology 2012 61 562 575 22765314 10.1111/j.1365-2559.2012.04270.x

[B24] Horcic M Koelzer VH Karamitopoulou E Terracciano L Puppa G Zlobec I Lugli A Tumour budding score based on 10 high-power fields is a promising basis for a standardised prognostic scoring system in stage II colorectal cancer Hum Pathol 2013 44 697 705 23159156 10.1016/j.humpath.2012.07.026

[B25] Hasan J Byers R Jayson GC Intra-tumoural microvessel density in human solid tumours Br J Cancer 2002 86 1566 1577 12085206 10.1038/sj.bjc.6600315 PMC2746601

[B26] Roxburgh CS McMillan DC Anderson JH McKee RF Horgan PG Foulis AK Elastica staining for venous invasion results in superior prediction of cancer-specific survival in colorectal cancer Ann Surg 2010 252 989 997 21107109 10.1097/SLA.0b013e3181f1c60d

[B27] Kingston EF Goulding H Bateman AC Vascular invasion is underrecognized in colorectal cancer using conventional hematoxylin and eosin staining Dis Colon Rectum 2007 50 1867 1872 17665249 10.1007/s10350-007-9021-6

[B28] Ohtsuki K Koyama F Tamura T Enomoto Y Fujii H Mukogawa T Nakagawa T Uchimoto K Nakamura S Nonomura A Nakajima Y Prognostic value of immunohistochemical analysis of tumour budding in colorectal carcinoma Anticancer Res 2008 28 1831 1836 18630467

[B29] Lin M Ma SP Lin HZ Ji P Xie D Yu JX Intratumoural as well as peritumoural lymphatic vessel invasion correlates with lymph node metastasis and unfavourable outcome in colorectal cancer Clin Exp Metastasis 2010 27 123 132 20195706 10.1007/s10585-010-9309-0

[B30] Kazama S Watanabe T Ajioka Y Kanazawa T Nagawa H Tumour budding at the deepest invasive margin correlates with lymph node metastasis in submucosal colorectal cancer detected by anticytokeratin antibody CAM5.2 Br J Cancer 2006 94 293 298 16404429 10.1038/sj.bjc.6602927 PMC2361114

[B31] Tetu B Evans A Canadian Licensure for the Use of Digital Pathology for Routine Diagnoses Arch Pathol Lab Med 2013 138 302 304 23802851 10.5858/arpa.2013-0289-ED

[B32] Pages F Berger A Camus M Sanchez-Cabo F Costes A Molidor R Mlecnik B Kirilovsky A Nilsson M Damotte D Meatchi T Bruneval P Cugnenc PH Trajanoski Z Fridman WH Galon J Effector memory T cells, early metastasis, and survival in colorectal cancer N Engl J Med 2005 353 2654 2666 16371631 10.1056/NEJMoa051424

[B33] Angell HK Gray N Womack C Pritchard DI Wilkinson RW Cumberbatch M Digital pattern recognition-based image analysis quantifies immune infiltrates in distinct tissue regions of colorectal cancer and identifies a metastatic phenotype Br J Cancer 2013 109 1618 1624 23963148 10.1038/bjc.2013.487 PMC3776996

[B34] Hase K Shatney C Johnson D Trollope M Vierra M Prognostic value of tumour "budding" in patients with colorectal cancer Dis Colon Rectum 1993 36 627 635 8348847 10.1007/BF02238588

[B35] Camp RL Dolled-Filhart M Rimm DL X-tile: a new bio-informatics tool for biomarker assessment and outcome-based cut-point optimization Clin Cancer Res 2004 10 7252 7259 15534099 10.1158/1078-0432.CCR-04-0713

[B36] Lubbock AL Katz E Harrison DJ Overton IM TMA Navigator: Network inference, patient stratification and survival analysis with tissue microarray data Nucleic Acids Res 2013 41 W562 W568 23761446 10.1093/nar/gkt529 PMC3692046

[B37] Midgley R Kerr DJ Adjuvant chemotherapy for stage II colorectal cancer: the time is right! Nat Clin Pract Oncol 2005 2 364 369 16075796 10.1038/ncponc0228

[B38] Wang TB Chen ZG Wei XQ Wei B Dong WG Serum vascular endothelial growth factor-C and lymphoangiogenesis are associated with the lymph node metastasis and prognosis of patients with colorectal cancer ANZ J Surg 2011 81 694 699 22295309 10.1111/j.1445-2197.2010.05539.x

[B39] Gao J Knutsen A Arbman G Carstensen J Franlund B Sun XF Clinical and biological significance of angiogenesis and lymphangiogenesis in colorectal cancer Dig Liver Dis 2009 41 116 122 19038587 10.1016/j.dld.2008.07.315

[B40] Longatto-Filho A Pinheiro C Ferreira L Scapulatempo C Alves VA Baltazar F Schmitt F Peritumoural, but not intratumoural, lymphatic vessel density and invasion correlate with colorectal carcinoma poor-outcome markers Virchows Arch 2008 452 133 138 18087718 10.1007/s00428-007-0550-0

[B41] Matsumoto K Nakayama Y Inoue Y Minagawa N Katsuki T Shibao K Tsurudome Y Hirata K Nagata N Itoh H Lymphatic microvessel density is an independent prognostic factor in colorectal cancer Dis Colon Rectum 2007 50 308 314 17164964 10.1007/s10350-006-0792-y

