# Supplementary figures and images for: Quantification of tumour budding, lymphatic vessel density and invasion through image analysis in colorectal cancer
Source: J Transl Med. 2014 Jun 1;12:156. doi: 10.1186/1479-5876-12-156 (PMC4098951; doi:10.1186/1479-5876-12-156)

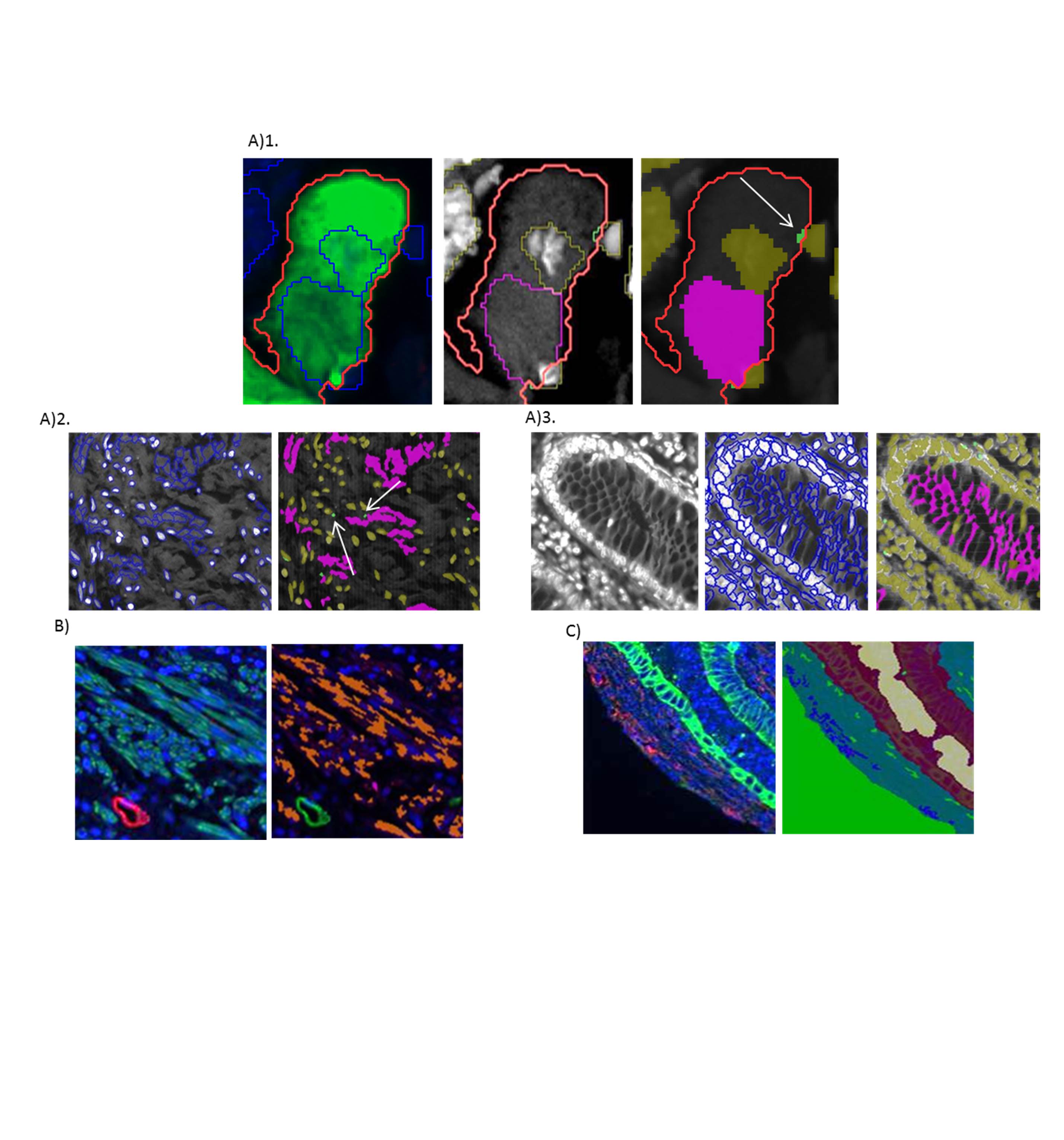

Supplement: Additional file 1: Figure S1 — Classification of false nuclei and objects. Original composite images are pseudocoloured blue (DAPI), green (panCK) and red (D2-40). Images for the DAPI channel alone are greyscale. A)1. False nuclei are classified and negated as are nuclear debris. To negate a false nuclei count, post-segmentation, in the stroma or within tumour buds, nuclei under 16 μm2 are classified as debris nuclei. A white arrow shows a small section from a stromal nucleus being segmented by the tumour bud and classified as debris nucleus. A high background intensity of DAPI, increased by a segmented section of a stromal nucleus within the tumour bud, has created a false nucleus (pink). This is classified as such and negated. A)2. The tissue section in the example has high auto-fluorescence within the DAPI channel, the false nuclei segmented as a result of this are classified as such (pink). A)3. High DAPI channel auto-fluorescence of goblet cells within colonic crypts result in false nuclei segmentation, these are too classified as such (pink). B) Auto-fluorescence of muscle cells, within the Cy3 channel, may lead to their segmentation as panCk positive epithelial cells. Intensity and texture parameters are utilised to classify the falsely segmented objects as ‘non-specific CK’. C) Non-specific staining of both panCk and D2-40 antibodies occurs close to the edge of tissue. All positively segmented objects which are 50 μm from ‘no tissue’ (green) are classified as ‘edge effect’ (blue). [file 1479-5876-12-156-S1.jpeg]

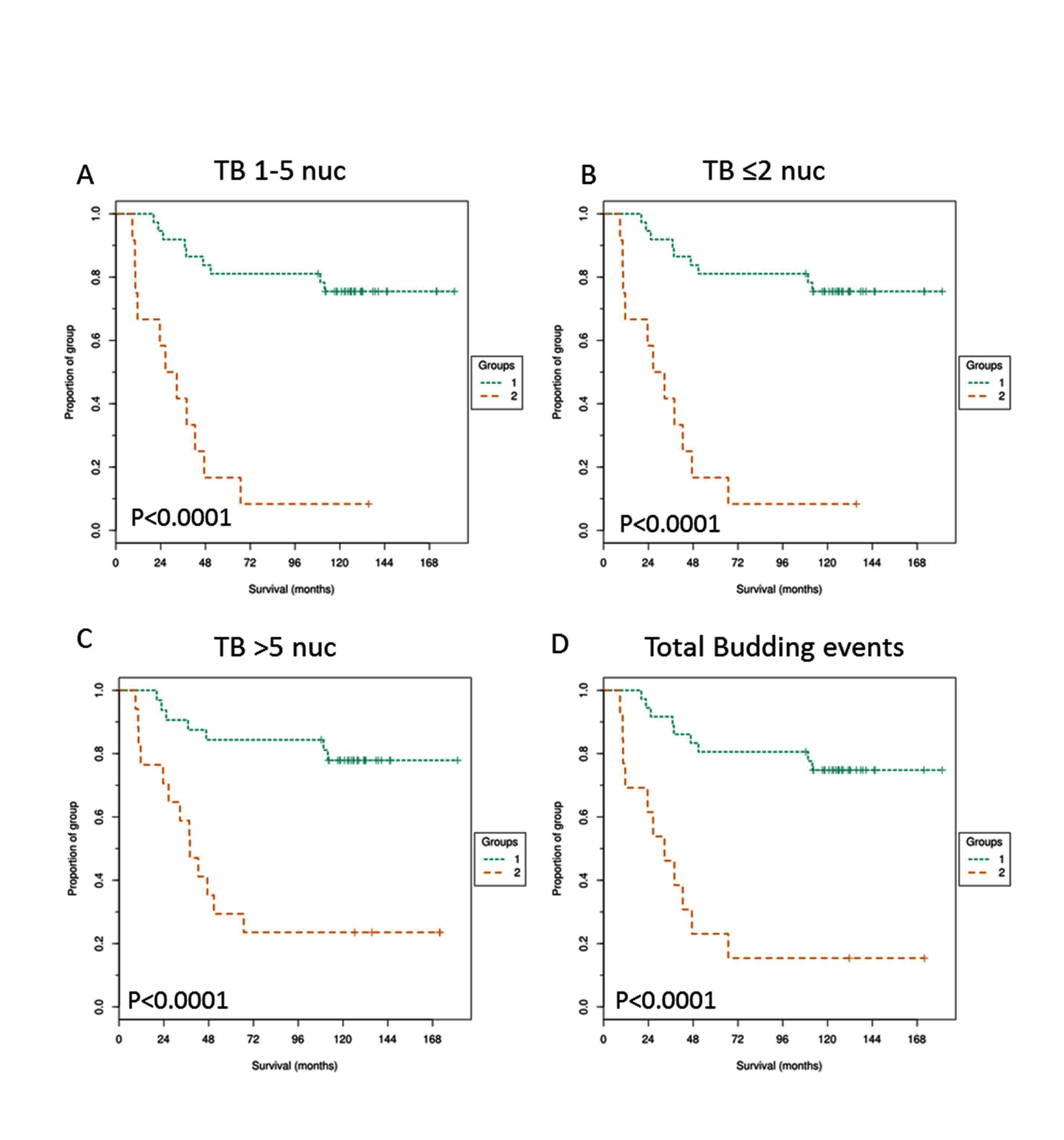

Supplement: Additional file 2: Figure S2 — Kaplan-Meier plots comparing prognostic significance of quantifying differing sizes of tumour bud. A) Kaplan-Meier curve showing disease specific survival times for below cut-off (group 1) and above cut-off (group 2) in tumour buds with 1–5 nuclei associated. B) Kaplan-Meier curve showing disease specific survival times for below cut-off (group 1) and above cut-off (group 2)in tumour buds with 1–2 nuclei associated. C) Kaplan-Meier curve showing disease specific survival times for below cut-off (group 1) and above cut-off (group 2) in tumour buds with greater than 5 nuclei associated. D) Kaplan-Meier curve showing disease specific survival times for below cut-off (group 1) and above cut-off (group 2) upon the summing of tumour buds with 1–5 nuclei and tumour buds with greater than 5 nuclei. [file 1479-5876-12-156-S2.jpeg]
